# Supplementary material for: Trends in childhood cancer: Incidence and survival analysis over 45 years of SEER data
Source: PLoS One. 2025 Jan 3;20(1):e0314592. doi: 10.1371/journal.pone.0314592 (PMC11698462; doi:10.1371/journal.pone.0314592)
Supplement: S1 Table — (DOCX) [file pone.0314592.s001.docx]

| **S1 table.** Yearly Trends in Childhood Cancer Rates, Stratified by Race, Age Group, and Sex, as Derived from SEER*Stat Trends Analysis (Darker Color indicates Higher APC). | | | | | | |
| --- | --- | --- | --- | --- | --- | --- |
| **Race** | **Sex** | **ICCC** | **APC** | **P** | **Lower CI** | **Upper CI** |
| White | F | I LEUKEMIAS, MYELOPROLIFERATIVE AND MYELODYSPLASTIC DISEASES | 0.93 | 0.00 | 0.67 | 1.20 |
| Black | M | I LEUKEMIAS, MYELOPROLIFERATIVE AND MYELODYSPLASTIC DISEASES | 0.93 | 0.00 | 0.35 | 1.52 |
| White | Both | I LEUKEMIAS, MYELOPROLIFERATIVE AND MYELODYSPLASTIC DISEASES | 0.91 | 0.00 | 0.72 | 1.10 |
| White | M | I LEUKEMIAS, MYELOPROLIFERATIVE AND MYELODYSPLASTIC DISEASES | 0.89 | 0.00 | 0.64 | 1.13 |
| All races | M | I LEUKEMIAS, MYELOPROLIFERATIVE AND MYELODYSPLASTIC DISEASES | 0.85 | 0.00 | 0.65 | 1.04 |
| All races | Both | I LEUKEMIAS, MYELOPROLIFERATIVE AND MYELODYSPLASTIC DISEASES | 0.84 | 0.00 | 0.68 | 1.00 |
| All races | F | I LEUKEMIAS, MYELOPROLIFERATIVE AND MYELODYSPLASTIC DISEASES | 0.82 | 0.00 | 0.59 | 1.05 |
| Black | Both | I LEUKEMIAS, MYELOPROLIFERATIVE AND MYELODYSPLASTIC DISEASES | 0.55 | 0.02 | 0.11 | 0.99 |
| Other (American Indian/AK Native, Asian/Pacific Isl&er) | Both | II LYMPHOMAS AND RETICULOENDOTHELIAL NEOPLASMS | 2.03 | 0.00 | 1.35 | 2.72 |
| Black | Both | II LYMPHOMAS AND RETICULOENDOTHELIAL NEOPLASMS | 1.34 | 0.00 | 0.72 | 1.97 |
| All races | M | II LYMPHOMAS AND RETICULOENDOTHELIAL NEOPLASMS | 0.76 | 0.00 | 0.43 | 1.09 |
| Black | M | II LYMPHOMAS AND RETICULOENDOTHELIAL NEOPLASMS | 0.75 | 0.05 | -0.02 | 1.53 |
| All races | Both | II LYMPHOMAS AND RETICULOENDOTHELIAL NEOPLASMS | 0.72 | 0.00 | 0.45 | 0.98 |
| All races | F | II LYMPHOMAS AND RETICULOENDOTHELIAL NEOPLASMS | 0.64 | 0.00 | 0.33 | 0.94 |
| White | M | II LYMPHOMAS AND RETICULOENDOTHELIAL NEOPLASMS | 0.57 | 0.00 | 0.21 | 0.94 |
| White | Both | II LYMPHOMAS AND RETICULOENDOTHELIAL NEOPLASMS | 0.53 | 0.00 | 0.25 | 0.81 |
| White | F | III CNS AND MISCELLANEOUS INTRACRANIAL AND INTRASPINAL NEOPLASMS | 0.92 | 0.00 | 0.53 | 1.32 |
| White | Both | III CNS AND MISCELLANEOUS INTRACRANIAL AND INTRASPINAL NEOPLASMS | 0.83 | 0.00 | 0.57 | 1.10 |
| All races | F | III CNS AND MISCELLANEOUS INTRACRANIAL AND INTRASPINAL NEOPLASMS | 0.81 | 0.00 | 0.47 | 1.15 |
| Other (American Indian/AK Native, Asian/Pacific Isl&er) | F | III CNS AND MISCELLANEOUS INTRACRANIAL AND INTRASPINAL NEOPLASMS | 0.76 | 0.11 | -0.18 | 1.72 |
| White | M | III CNS AND MISCELLANEOUS INTRACRANIAL AND INTRASPINAL NEOPLASMS | 0.74 | 0.00 | 0.48 | 1.01 |
| All races | Both | III CNS AND MISCELLANEOUS INTRACRANIAL AND INTRASPINAL NEOPLASMS | 0.71 | 0.00 | 0.45 | 0.97 |
| All races | M | III CNS AND MISCELLANEOUS INTRACRANIAL AND INTRASPINAL NEOPLASMS | 0.61 | 0.00 | 0.33 | 0.89 |
| White | F | IIIa Ependymomas & choroid plexus tumor | 1.38 | 0.00 | 0.55 | 2.22 |
| All races | F | IIIa Ependymomas & choroid plexus tumor | 1.20 | 0.00 | 0.41 | 2.00 |
| White | Both | IIIa Ependymomas & choroid plexus tumor | 1.07 | 0.00 | 0.49 | 1.66 |
| All races | Both | IIIa Ependymomas & choroid plexus tumor | 0.99 | 0.00 | 0.44 | 1.53 |
| White | M | IIIa Ependymomas & choroid plexus tumor | 0.70 | 0.09 | -0.11 | 1.52 |
| All races | M | IIIa Ependymomas & choroid plexus tumor | 0.67 | 0.08 | -0.08 | 1.43 |
| White | F | IIIa1 Ependymomas | 1.28 | 0.01 | 0.35 | 2.22 |
| All races | F | IIIa1 Ependymomas | 1.08 | 0.01 | 0.22 | 1.95 |
| White | Both | IIIa1 Ependymomas | 0.84 | 0.02 | 0.14 | 1.54 |
| All races | Both | IIIa1 Ependymomas | 0.76 | 0.02 | 0.12 | 1.40 |
| White | F | IIIb Astrocytomas | 0.84 | 0.00 | 0.32 | 1.37 |
| White | Both | IIIb Astrocytomas | 0.77 | 0.00 | 0.42 | 1.12 |
| All races | F | IIIb Astrocytomas | 0.74 | 0.01 | 0.23 | 1.25 |
| White | M | IIIb Astrocytomas | 0.71 | 0.00 | 0.37 | 1.05 |
| All races | Both | IIIb Astrocytomas | 0.66 | 0.00 | 0.30 | 1.01 |
| Other (American Indian/AK Native, Asian/Pacific Isl&er) | Both | IIIb Astrocytomas | 0.61 | 0.26 | -0.45 | 1.68 |
| All races | M | IIIb Astrocytomas | 0.58 | 0.00 | 0.24 | 0.91 |
| White | F | IIIc Intracranial & intraspinal embryonal tumors | 0.92 | 0.01 | 0.22 | 1.63 |
| All races | F | IIIc Intracranial & intraspinal embryonal tumors | 0.88 | 0.01 | 0.27 | 1.49 |
| White | Both | IIIc Intracranial & intraspinal embryonal tumors | 0.81 | 0.00 | 0.34 | 1.29 |
| All races | Both | IIIc Intracranial & intraspinal embryonal tumors | 0.74 | 0.00 | 0.31 | 1.18 |
| White | M | IIIc Intracranial & intraspinal embryonal tumors | 0.72 | 0.02 | 0.11 | 1.33 |
| All races | M | IIIc Intracranial & intraspinal embryonal tumors | 0.59 | 0.04 | 0.04 | 1.15 |
| White | F | IIId Other gliomas | 1.19 | 0.00 | 0.48 | 1.90 |
| All races | F | IIId Other gliomas | 1.07 | 0.00 | 0.38 | 1.76 |
| White | Both | IIId Other gliomas | 0.99 | 0.00 | 0.43 | 1.55 |
| All races | Both | IIId Other gliomas | 0.82 | 0.00 | 0.32 | 1.33 |
| White | M | IIId Other gliomas | 0.81 | 0.03 | 0.07 | 1.57 |
| All races | M | IIId Other gliomas | 0.60 | 0.07 | -0.06 | 1.27 |
| White | M | IIId2 Mixed & unspecified gliomas | 1.70 | 0.00 | 0.97 | 2.43 |
| White | Both | IIId2 Mixed & unspecified gliomas | 1.67 | 0.00 | 1.09 | 2.25 |
| White | F | IIId2 Mixed & unspecified gliomas | 1.64 | 0.00 | 0.85 | 2.43 |
| All races | F | IIId2 Mixed & unspecified gliomas | 1.49 | 0.00 | 0.79 | 2.18 |
| All races | Both | IIId2 Mixed & unspecified gliomas | 1.44 | 0.00 | 0.94 | 1.94 |
| All races | M | IIId2 Mixed & unspecified gliomas | 1.32 | 0.00 | 0.62 | 2.03 |
| All races | F | IIb NHL (except Burkitt lymphoma) | 2.07 | 0.00 | 1.50 | 2.63 |
| White | F | IIb NHL (except Burkitt lymphoma) | 1.89 | 0.00 | 1.22 | 2.57 |
| All races | Both | IIb NHL (except Burkitt lymphoma) | 1.70 | 0.00 | 1.36 | 2.04 |
| White | Both | IIb NHL (except Burkitt lymphoma) | 1.49 | 0.00 | 1.14 | 1.83 |
| All races | M | IIb NHL (except Burkitt lymphoma) | 1.46 | 0.00 | 1.00 | 1.93 |
| White | M | IIb NHL (except Burkitt lymphoma) | 1.16 | 0.00 | 0.69 | 1.63 |
| All races | M | IIb2 Mature B-cell lymphomas (except Burkitt lymphoma) | 0.97 | 0.01 | 0.27 | 1.67 |
| All races | Both | IIb2 Mature B-cell lymphomas (except Burkitt lymphoma) | 0.93 | 0.00 | 0.40 | 1.47 |
| All races | F | IIb2 Mature B-cell lymphomas (except Burkitt lymphoma) | 0.74 | 0.09 | -0.13 | 1.63 |
| White | M | IIb2 Mature B-cell lymphomas (except Burkitt lymphoma) | 0.64 | 0.07 | -0.06 | 1.34 |
| White | Both | IIb2 Mature B-cell lymphomas (except Burkitt lymphoma) | 0.63 | 0.02 | 0.09 | 1.16 |
| All races | M | IX SOFT TISSUE AND OTHER EXTRAOSSEOUS SARCOMAS | 0.61 | 0.00 | 0.21 | 1.01 |
| White | M | IX SOFT TISSUE AND OTHER EXTRAOSSEOUS SARCOMAS | 0.55 | 0.02 | 0.08 | 1.02 |
| Black | Both | IX SOFT TISSUE AND OTHER EXTRAOSSEOUS SARCOMAS | 0.50 | 0.23 | -0.32 | 1.33 |
| White | M | IXd Other specified STSs | 1.23 | 0.00 | 0.48 | 1.98 |
| All races | M | IXd Other specified STSs | 1.11 | 0.00 | 0.46 | 1.76 |
| White | Both | IXd Other specified STSs | 0.99 | 0.00 | 0.39 | 1.59 |
| All races | Both | IXd Other specified STSs | 0.96 | 0.00 | 0.41 | 1.51 |
| Other (American Indian/AK Native, Asian/Pacific Isl&er) | Both | IXd Other specified STSs | 0.77 | 0.24 | -0.53 | 2.08 |
| All races | F | IXd Other specified STSs | 0.71 | 0.06 | -0.03 | 1.46 |
| White | F | IXd Other specified STSs | 0.57 | 0.19 | -0.29 | 1.45 |
| All races | M | IXe Unspecified soft tissue sarcomas | 2.01 | 0.00 | 0.79 | 3.24 |
| All races | Both | IXe Unspecified soft tissue sarcomas | 1.26 | 0.01 | 0.39 | 2.13 |
| White | F | Ia Lymphoid leukemias | 0.87 | 0.00 | 0.58 | 1.16 |
| White | Both | Ia Lymphoid leukemias | 0.84 | 0.00 | 0.64 | 1.05 |
| White | M | Ia Lymphoid leukemias | 0.81 | 0.00 | 0.54 | 1.08 |
| All races | F | Ia Lymphoid leukemias | 0.71 | 0.00 | 0.45 | 0.97 |
| All races | Both | Ia Lymphoid leukemias | 0.69 | 0.00 | 0.50 | 0.88 |
| All races | M | Ia Lymphoid leukemias | 0.67 | 0.00 | 0.45 | 0.88 |
| White | F | Ia1 Precursor cell leukemias | 0.83 | 0.00 | 0.53 | 1.12 |
| White | Both | Ia1 Precursor cell leukemias | 0.80 | 0.00 | 0.58 | 1.01 |
| White | M | Ia1 Precursor cell leukemias | 0.76 | 0.00 | 0.48 | 1.04 |
| All races | F | Ia1 Precursor cell leukemias | 0.66 | 0.00 | 0.40 | 0.93 |
| All races | Both | Ia1 Precursor cell leukemias | 0.64 | 0.00 | 0.45 | 0.84 |
| All races | M | Ia1 Precursor cell leukemias | 0.62 | 0.00 | 0.39 | 0.85 |
| All races | M | Ib AMLs | 0.98 | 0.00 | 0.48 | 1.48 |
| All races | Both | Ib AMLs | 0.88 | 0.00 | 0.49 | 1.28 |
| White | F | Ib AMLs | 0.81 | 0.03 | 0.10 | 1.53 |
| White | Both | Ib AMLs | 0.79 | 0.00 | 0.34 | 1.24 |
| White | M | Ib AMLs | 0.76 | 0.01 | 0.20 | 1.33 |
| All races | F | Ib AMLs | 0.73 | 0.01 | 0.17 | 1.30 |
| All races | Both | Ic Chronic myeloproliferative diseases | 2.42 | 0.00 | 1.55 | 3.31 |
| All races | M | Ic Chronic myeloproliferative diseases | 2.25 | 0.00 | 1.28 | 3.23 |
| White | Both | Ic Chronic myeloproliferative diseases | 2.20 | 0.00 | 1.19 | 3.21 |
| White | F | V RETINOBLASTOMA | 0.50 | 0.23 | -0.33 | 1.34 |
| All races | Both | VII HEPATIC TUMORS | 2.17 | 0.00 | 1.45 | 2.89 |
| All races | M | VII HEPATIC TUMORS | 2.16 | 0.00 | 1.15 | 3.18 |
| White | M | VII HEPATIC TUMORS | 2.09 | 0.00 | 0.98 | 3.21 |
| White | Both | VII HEPATIC TUMORS | 2.06 | 0.00 | 1.25 | 2.88 |
| All races | F | VII HEPATIC TUMORS | 1.93 | 0.00 | 1.08 | 2.79 |
| White | F | VII HEPATIC TUMORS | 1.87 | 0.00 | 0.87 | 2.88 |
| White | F | VIIIa Osteosarcomas | 0.86 | 0.02 | 0.17 | 1.55 |
| White | Both | VIIIa Osteosarcomas | 0.84 | 0.00 | 0.34 | 1.33 |
| White | M | VIIIa Osteosarcomas | 0.74 | 0.07 | -0.05 | 1.54 |
| All races | Both | VIIIa Osteosarcomas | 0.54 | 0.02 | 0.09 | 1.00 |
| All races | F | VIIIa Osteosarcomas | 0.54 | 0.10 | -0.11 | 1.19 |
| All races | Both | VIIa Hepatoblastoma & mesenchymal tumors of liver | 2.46 | 0.00 | 1.68 | 3.25 |
| White | Both | VIIa Hepatoblastoma & mesenchymal tumors of liver | 2.20 | 0.00 | 1.29 | 3.13 |
| All races | Both | VIIa1 Hepatoblastoma | 2.31 | 0.00 | 1.46 | 3.17 |
| White | Both | VIIa1 Hepatoblastoma | 1.99 | 0.00 | 1.02 | 2.96 |
| White | M | X GERM CELL TUMORS, TROPHOBLASTIC TUMORS AND NEOPLASMS OF GONADS | 0.83 | 0.00 | 0.34 | 1.32 |
| All races | M | X GERM CELL TUMORS, TROPHOBLASTIC TUMORS AND NEOPLASMS OF GONADS | 0.67 | 0.00 | 0.23 | 1.11 |
| Other (American Indian/AK Native, Asian/Pacific Isl&er) | M | X GERM CELL TUMORS, TROPHOBLASTIC TUMORS AND NEOPLASMS OF GONADS | 0.63 | 0.24 | -0.42 | 1.68 |
| White | Both | X GERM CELL TUMORS, TROPHOBLASTIC TUMORS AND NEOPLASMS OF GONADS | 0.62 | 0.00 | 0.28 | 0.96 |
| All races | Both | X GERM CELL TUMORS, TROPHOBLASTIC TUMORS AND NEOPLASMS OF GONADS | 0.50 | 0.00 | 0.19 | 0.81 |
| White | F | XI OTHER MALIGNANT EPITHELIAL NEOPLASMS AND MALIGNANT MELANOMAS | 1.85 | 0.00 | 1.50 | 2.20 |
| White | Both | XI OTHER MALIGNANT EPITHELIAL NEOPLASMS AND MALIGNANT MELANOMAS | 1.83 | 0.00 | 1.55 | 2.11 |
| White | M | XI OTHER MALIGNANT EPITHELIAL NEOPLASMS AND MALIGNANT MELANOMAS | 1.74 | 0.00 | 1.30 | 2.18 |
| All races | F | XI OTHER MALIGNANT EPITHELIAL NEOPLASMS AND MALIGNANT MELANOMAS | 1.62 | 0.00 | 1.29 | 1.95 |
| All races | Both | XI OTHER MALIGNANT EPITHELIAL NEOPLASMS AND MALIGNANT MELANOMAS | 1.59 | 0.00 | 1.32 | 1.86 |
| All races | M | XI OTHER MALIGNANT EPITHELIAL NEOPLASMS AND MALIGNANT MELANOMAS | 1.52 | 0.00 | 1.12 | 1.91 |
| White | M | XIb Thyroid carcinomas | 2.70 | 0.00 | 1.75 | 3.65 |
| White | Both | XIb Thyroid carcinomas | 2.59 | 0.00 | 2.11 | 3.08 |
| White | F | XIb Thyroid carcinomas | 2.57 | 0.00 | 2.05 | 3.10 |
| All races | Both | XIb Thyroid carcinomas | 2.40 | 0.00 | 1.99 | 2.82 |
| All races | F | XIb Thyroid carcinomas | 2.39 | 0.00 | 1.92 | 2.87 |
| All races | M | XIb Thyroid carcinomas | 2.34 | 0.00 | 1.50 | 3.19 |
| White | M | XId Malignant melanomas | 0.75 | 0.09 | -0.11 | 1.63 |
| White | Both | XId Malignant melanomas | 0.73 | 0.01 | 0.19 | 1.27 |
| White | F | XId Malignant melanomas | 0.60 | 0.09 | -0.10 | 1.32 |
| All races | M | XIf Other & unspecified carcinomas | 2.45 | 0.00 | 1.80 | 3.11 |
| White | M | XIf Other & unspecified carcinomas | 2.45 | 0.00 | 1.73 | 3.17 |
| White | Both | XIf Other & unspecified carcinomas | 2.36 | 0.00 | 1.69 | 3.03 |
| All races | Both | XIf Other & unspecified carcinomas | 2.15 | 0.00 | 1.50 | 2.81 |
| White | F | XIf Other & unspecified carcinomas | 2.14 | 0.00 | 1.30 | 3.00 |
| All races | F | XIf Other & unspecified carcinomas | 1.77 | 0.00 | 0.94 | 2.62 |
| White | Both | XIf10 Carcinomas of other specified sites | 2.06 | 0.00 | 1.14 | 2.99 |
| All races | Both | XIf10 Carcinomas of other specified sites | 1.93 | 0.00 | 1.16 | 2.71 |
| All races | Both | Xa Intracranial & intraspinal GCTs | 1.18 | 0.01 | 0.27 | 2.09 |
| All races | Both | Xa1 Intracranial & intraspinal germinomas | 1.65 | 0.00 | 0.55 | 2.77 |
| White | F | Xb Malignant extracranial & extragonadal GCTs | 0.66 | 0.18 | -0.31 | 1.65 |
| White | M | Xc Malignant gonadal GCTs | 0.86 | 0.00 | 0.37 | 1.35 |
| White | Both | Xc Malignant gonadal GCTs | 0.70 | 0.00 | 0.30 | 1.11 |
| All races | M | Xc Malignant gonadal GCTs | 0.60 | 0.01 | 0.15 | 1.06 |
| White | Both | Xc1 Malignant gonadal germinomas | 1.73 | 0.00 | 0.84 | 2.62 |
| All races | Both | Xc1 Malignant gonadal germinomas | 1.19 | 0.01 | 0.30 | 2.08 |

Abbreviations: AMLs, acute myeloid leukemias; APC, annual percent change; CI, confidence interval; F, female; GCTs, germ cell tumors; ICCC, International Classification of Childhood Cancer; M, male; NHL, non-Hodgkin lymphoma; STSs, soft-tissue sarcomas
