# Supplementary material for: Trends in childhood cancer: Incidence and survival analysis over 45 years of SEER data
Source: PLoS One. 2025 Jan 3;20(1):e0314592. doi: 10.1371/journal.pone.0314592 (PMC11698462; doi:10.1371/journal.pone.0314592)
Supplement: S2 Table — (DOCX) [file pone.0314592.s002.docx]

S2 table. Distribution and Survival Rates of Childhood Cancers by Stage and Decade According to the International Classification of Childhood Cancer (ICCC)

| **ICCC** | **SEER stage** | **1976-1979** | | **1980-1989** | | **1990-1999** | | **2000-2009** | | **2010-2019** | |
| --- | --- | --- | --- | --- | --- | --- | --- | --- | --- | --- | --- |
|  |  | **N** | **5-year OS** | **N** | **5-year OS** | **N** | **5-year OS** | **N** | **5-year OS** | **N** | **5-year OS** |
| Leukemias | Localized | NA | NA | NA | NA | NA | NA | 8 (0.1%) | 37.5 | 19 (0.1%) | 100 |
| Lymphomas | Localized | NA | NA | NA | NA | NA | NA | 695 (6.5%) | 93.46 | 1949 (12.9%) | 97.64 |
| CNS | Localized | 4 (0.5%) | 66.67 | 14 (0.9%) | 50 | 32 (1.3%) | 59.38 | 3184 (29.6%) | 77.15 | 5289 (35%) | 77.62 |
| Neuroblastoma | Localized | 40 (5.1%) | 92.11 | 70 (4.5%) | 92.54 | 125 (5%) | 93.41 | 367 (3.4%) | 97.72 | 422 (2.8%) | 96.15 |
| Retinoblastoma | Localized | 59 (7.5%) | 98.31 | 128 (8.2%) | 95.31 | 231 (9.2%) | 97.81 | 524 (4.9%) | 98.45 | 542 (3.6%) | 98.58 |
| Renal tumors | Localized | 83 (10.5%) | 86.59 | 179 (11.5%) | 96.62 | 242 (9.6%) | 93.34 | 624 (5.8%) | 95.13 | 604 (4%) | 98.17 |
| Hepatic tumors | Localized | 12 (1.5%) | 33.33 | 26 (1.7%) | 76.92 | 63 (2.5%) | 72.92 | 223 (2.1%) | 83.25 | 306 (2%) | 89.75 |
| Bone tumors | Localized | 94 (11.9%) | 48.94 | 135 (8.6%) | 70.9 | 234 (9.3%) | 79.77 | 634 (5.9%) | 83.34 | 750 (5%) | 86.45 |
| STS | Localized | 138 (17.5%) | 82.48 | 273 (17.5%) | 88.15 | 401 (16%) | 89.72 | 1146 (10.6%) | 89.26 | 1301 (8.6%) | 91.63 |
| GCT | Localized | 121 (15.4%) | 81.82 | 282 (18.1%) | 93.23 | 496 (19.7%) | 95.95 | 1190 (11.1%) | 96.41 | 1143 (7.6%) | 95.23 |
| Other | Localized | 235 (29.9%) | 93.47 | 445 (28.5%) | 96.61 | 677 (26.9%) | 96.59 | 2110 (19.6%) | 98.28 | 2702 (17.9%) | 98.73 |
| Unspecified | Localized | 1 (0.1%) | 100 | 10 (0.6%) | 80 | 13 (0.5%) | 100 | 61 (0.6%) | 91.55 | 65 (0.4%) | 91.25 |
| Leukemias | Regional | NA | NA | NA | NA | NA | NA | NA | NA | 1 (0%) | 100 |
| Lymphomas | Regional | NA | NA | NA | NA | NA | NA | 1112 (19%) | 93.96 | 1673 (24.4%) | 96.58 |
| CNS | Regional | NA | NA | 6 (0.7%) | 83.33 | 29 (1.8%) | 62.07 | 482 (8.2%) | 58.79 | 658 (9.6%) | 63.23 |
| Neuroblastoma | Regional | 31 (8.6%) | 64.52 | 95 (11.1%) | 79.62 | 194 (12.3%) | 85.52 | 419 (7.1%) | 92.74 | 368 (5.4%) | 94.1 |
| Retinoblastoma | Regional | 7 (1.9%) | 71.43 | 14 (1.6%) | 85.71 | 18 (1.1%) | 88.89 | 108 (1.8%) | 93.48 | 91 (1.3%) | 85.33 |
| Renal tumors | Regional | 37 (10.3%) | 70.27 | 105 (12.2%) | 85.7 | 194 (12.3%) | 88.14 | 403 (6.9%) | 92.72 | 469 (6.8%) | 93.31 |
| Hepatic tumors | Regional | 13 (3.6%) | 23.08 | 29 (3.4%) | 48.28 | 60 (3.8%) | 55 | 152 (2.6%) | 63.98 | 187 (2.7%) | 76.63 |
| Bone tumors | Regional | 69 (19.2%) | 56.52 | 177 (20.6%) | 63.28 | 283 (17.9%) | 61.96 | 832 (14.2%) | 71.89 | 629 (9.2%) | 75.77 |
| STS | Regional | 62 (17.3%) | 62.9 | 128 (14.9%) | 72.66 | 288 (18.3%) | 76.55 | 758 (12.9%) | 73.11 | 592 (8.6%) | 74.22 |
| GCT | Regional | 37 (10.3%) | 64.86 | 105 (12.2%) | 89.52 | 147 (9.3%) | 93.19 | 451 (7.7%) | 91.06 | 465 (6.8%) | 91.08 |
| Other | Regional | 102 (28.4%) | 90.1 | 196 (22.8%) | 85.63 | 358 (22.7%) | 88.48 | 1124 (19.2%) | 92.78 | 1698 (24.8%) | 95.53 |
| Unspecified | Regional | 1 (0.3%) | 0 | 4 (0.5%) | 100 | 7 (0.4%) | 85.71 | 23 (0.4%) | 69.57 | 26 (0.4%) | 75.28 |
| Leukemias | Distant | 906 (72.3%) | 48.19 | 2053 (74%) | 62.02 | 3839 (76.4%) | 72.84 | 10388 (68.2%) | 80.6 | 10960 (63.4%) | 85.04 |
| Lymphomas | Distant | 1 (0.1%) | 0 | 1 (0%) | 0 | 2 (0%) | 100 | 1432 (9.4%) | 87.82 | 2844 (16.5%) | 90.7 |
| CNS | Distant | 1 (0.1%) | 0 | NA | NA | 18 (0.4%) | 22.22 | 280 (1.8%) | 52.12 | 399 (2.3%) | 52.29 |
| Neuroblastoma | Distant | 98 (7.8%) | 32.41 | 217 (7.8%) | 36.59 | 314 (6.2%) | 48.06 | 843 (5.5%) | 59.3 | 845 (4.9%) | 67.54 |
| Retinoblastoma | Distant | 4 (0.3%) | 100 | 13 (0.5%) | 84.62 | 39 (0.8%) | 87.18 | 50 (0.3%) | 94 | 7 (0%) | 28.57 |
| Renal tumors | Distant | 43 (3.4%) | 46.51 | 91 (3.3%) | 72.19 | 140 (2.8%) | 74.21 | 323 (2.1%) | 71.98 | 346 (2%) | 77.87 |
| Hepatic tumors | Distant | 8 (0.6%) | 12.5 | 23 (0.8%) | 21.74 | 52 (1%) | 33.33 | 155 (1%) | 44.11 | 128 (0.7%) | 51.28 |
| Bone tumors | Distant | 41 (3.3%) | 26.83 | 96 (3.5%) | 15.62 | 143 (2.8%) | 39.86 | 434 (2.8%) | 39.02 | 525 (3%) | 44.23 |
| STS | Distant | 39 (3.1%) | 23.08 | 98 (3.5%) | 33.35 | 178 (3.5%) | 32.02 | 568 (3.7%) | 34.2 | 558 (3.2%) | 32.78 |
| GCT | Distant | 76 (6.1%) | 61.84 | 124 (4.5%) | 67.74 | 186 (3.7%) | 78.18 | 429 (2.8%) | 77.21 | 321 (1.9%) | 80.24 |
| Other | Distant | 33 (2.6%) | 50 | 56 (2%) | 55.36 | 109 (2.2%) | 52.17 | 311 (2%) | 52.6 | 319 (1.8%) | 57.68 |
| Unspecified | Distant | 3 (0.2%) | 66.67 | 4 (0.1%) | 33.33 | 8 (0.2%) | 15.62 | 25 (0.2%) | 58.43 | 27 (0.2%) | 68.33 |
| Leukemias | Unknown/in situ | NA | NA | NA | NA | 1 (0%) | 0 | 120 (1.9%) | 79.54 | 49 (2.2%) | 94.57 |
| Lymphomas | Unknown/in situ | 706 (44.4%) | 73.01 | 1359 (42.3%) | 78.55 | 1945 (39.4%) | 86.19 | 2178 (33.8%) | 87.7 | 296 (13.1%) | 92.93 |
| CNS | Unknown/in situ | 644 (40.5%) | 58.67 | 1392 (43.4%) | 64.69 | 2397 (48.6%) | 70.53 | 2722 (42.2%) | 72.06 | 333 (14.7%) | 77.91 |
| Neuroblastoma | Unknown/in situ | 45 (2.8%) | 50 | 94 (2.9%) | 46.15 | 84 (1.7%) | 69.07 | 140 (2.2%) | 72.18 | 93 (4.1%) | 88.14 |
| Retinoblastoma | Unknown/in situ | 2 (0.1%) | 100 | 12 (0.4%) | 90.91 | 40 (0.8%) | 97.44 | 54 (0.8%) | 93.96 | 84 (3.7%) | 96.69 |
| Renal tumors | Unknown/in situ | 3 (0.2%) | 100 | 16 (0.5%) | 100 | 22 (0.4%) | 85 | 58 (0.9%) | 96.43 | 48 (2.1%) | 91.87 |
| Hepatic tumors | Unknown/in situ | 2 (0.1%) | 0 | 9 (0.3%) | 43.75 | 17 (0.3%) | 26.67 | 26 (0.4%) | 58.67 | 26 (1.1%) | 62.3 |
| Bone tumors | Unknown/in situ | 21 (1.3%) | 71.43 | 72 (2.2%) | 70.59 | 65 (1.3%) | 72 | 111 (1.7%) | 78.23 | 88 (3.9%) | 75.89 |
| STS | Unknown/in situ | 57 (3.6%) | 50.88 | 90 (2.8%) | 65.92 | 102 (2.1%) | 69.08 | 206 (3.2%) | 73.51 | 185 (8.2%) | 72.15 |
| GCT | Unknown/in situ | 25 (1.6%) | 58.33 | 69 (2.1%) | 66.7 | 143 (2.9%) | 73.76 | 569 (8.8%) | 88.28 | 790 (34.8%) | 94.03 |
| Other | Unknown/in situ | 66 (4.1%) | 76.77 | 82 (2.6%) | 78.7 | 95 (1.9%) | 82.38 | 217 (3.4%) | 82.3 | 231 (10.2%) | 89.47 |
| Unspecified | Unknown/in situ | 20 (1.3%) | 35 | 15 (0.5%) | 57.14 | 26 (0.5%) | 68.18 | 42 (0.7%) | 87.28 | 44 (1.9%) | 82.92 |
