# Supplementary material for: Trends in childhood cancer: Incidence and survival analysis over 45 years of SEER data
Source: PLoS One. 2025 Jan 3;20(1):e0314592. doi: 10.1371/journal.pone.0314592 (PMC11698462; doi:10.1371/journal.pone.0314592)
Supplement: Attachment — Submitted filename: Rebuttal.docx [file pone.0314592.s003.docx]

Dr. Cho-Hao Howard Lee, M.D.

Academic Editor

PLOS ONE

6/11/2024

Dear Dr. Lee,

We are pleased to resubmit our revised manuscript titled "Trends in Childhood Cancer: Incidence and Survival Analysis Over 45 Years of SEER Data" (Manuscript ID:  [PONE-D-24-00928] - [EMID:b28eb0b3ecaea572]) for consideration for publication in PLOS ONE. We greatly appreciate the insightful and constructive feedback you and the reviewers provided, which has significantly improved our manuscript.

Please find below point by point response to your comments.

**Editor Comments**

1. **PLOS ONE's style requirements** : This was followed as instructed.
2. **Data Availability Statement:**
   a. The following was added to the title page:
      Data availability: The data utilized in this study are available from the SEER (Surveillance, Epidemiology, and End Results) registry. Researchers can request access to the data directly from the SEER registry.
3. **Full ethics statement in the ‘Methods’ section :**
   a. Ethical Approval: Ethical approval was not required for this study. The Institutional Review Board (IRB) of King Hussein Cancer Center (KHCC) waived the need for ethical approval as the study posed minimal risk and utilized securely de-identified data
4. **Supplementary tables included in the manuscript file**: Noted and fixed.
5. **Reference list to ensure is complete and correct**: Followed as instructed

6. **Introduction - Overview of Key Advancements:**

- **Response**: Thank you for suggesting an overview of key advancements in pediatric cancer diagnosis and treatment. We will incorporate a concise paragraph of significant advancements over the study period, highlighting major milestones that have influenced diagnostic and therapeutic approaches.
- **Added paragraph**:
  Over the last fifty years, the landscape of pediatric oncology has undergone significant evolution, marked by the introduction and refinement of chemotherapy, spearheaded by collaborative groups across North America and Europe. These efforts have led to the development of more effective treatment regimens that optimize the use of established drugs, resulting in markedly improved outcomes for almost all types of pediatric cancer. Enhancements in supportive care have rendered intensive treatments more manageable. Advances in stem cell transplantation techniques have become pivotal in rescuing patients who do not respond to initial treatments. Diagnostic progress, including molecular stratification, detection of minimal residual disease, and sophisticated genetic profiling, has refined therapeutic approaches, allowing for more tailored and effective treatments. Improvements in imaging technologies, such as advanced CT scanners and the advent of nuclear scanning, have significantly improved the detection of metastatic disease. Surgical and radiation oncology techniques have also seen substantial advancements, improving the precision and efficacy of tumor resection and control. The introduction of targeted therapies and immunotherapies has opened new avenues for treating specific patient subsets, including those with acute lymphoblastic leukemia (ALL), high-risk neuroblastoma, relapsed Hodgkin lymphoma, and others, marking a shift towards precision medicine. The integration of multidisciplinary care teams has further optimized treatment outcomes and patient care, emphasizing the importance of a holistic approach in the management of pediatric cancers.

2. **Methods - Detailing Statistical Analysis:**
   - **Response**: We appreciate the call for a more detailed description of our statistical analyses, particularly the joinpoint regression analysis. In the revision, we will expand this section to include detailed explanations of the statistical methods used, ensuring clarity and enhancing reproducibility.
3. **Results - Disparities Across Different Racial and Ethnic Groups:**
   - **Response**: You've highlighted an essential aspect of our study. We added the following paragraph to our discussion:
   - Racial disparities noted in our analysis is not a new finding. Even in standardized Children’s Oncology Group trials where patients receive the same regimens, black children had worse outcomes [44]. This is evident for different cancer types reported in previous analysis of the SEER database, but not in patients treated at St. Jude Children’s Research Hospital, where overlapping outcomes for all cancers were noted regardless of cancer type [45], suggesting that socioeconomic factors, rather than true biologic factors, are responsible for racial survival disparities.
4. **Discussion - Exploration of Reasons for Incidence Increases:** **Response**: We agree that exploring the potential reasons behind the observed increases in certain cancers could enrich the discussion. The following section was added to our discussion: Lifestyle and environmental changes over recent decades, such as increased exposure to pollutants and carcinogens, may have contributed to the rising rates of pediatric cancers. For instance, certain pesticides and parental smoking have been linked to an increased risk of childhood leukemia.[44-46] Moreover, the prevalence of obesity and related metabolic changes might also play a role in this upward trend, with more evidence in young adults.[47] Genetic predispositions, coupled with these environmental factors, underline the complexity of cancer etiology, necessitating multifaceted approaches in both research and public health interventions to address this growing concern.
5. **Figure and Table References:**
   - **Response**: We will ensure that all figures and tables are correctly referenced in the text and adhere strictly to the journal's formatting guidelines.

**Reviewer Comments**

**Reviewer #1**

- **General Appreciation**:
  - **Response**: We are grateful for your positive feedback on our data analysis approach.

**Reviewer #2**

1. **Introduction - Treatment-related Toxicities**:
   - **Response**: Treatment-related toxicity is a crucial factor in the management of childhood cancer. Unfortunately, the SEER database offers limited insight into this issue, primarily recording second cancers (which may or may not be related to toxicity) and deaths from other causes (e.g., cardiovascular disease as noted on death certificates). Greater understanding could be achieved by linking SEER data with other resources, such as Medicare/Medicaid. Although this approach is intriguing, it may exceed the scope of our paper. We have included a comment in the discussion section to highlight this point and acknowledge its significance in pediatric oncology.
2. **Results - Clarification on Mortality Rates**:

- **Response**: The section was confusing indeed. We rephrased it to highlight that we are referring to cancer as a cause of death among all children. We believe the paragraph now is easier to understand:
- Mortality records showed significant reductions in mortality rates for all children across various demographics over the analyzed period (Fig. 2). The age-adjusted all-cause mortality rates for all children declined from 125.0 per 100,000 in 1975–1979 to 52.0 per 100,000 in 2010–2019, indicating a substantial improvement in child health outcomes. A similar trend was observed for cancer as a cause of death among children, with the age-adjusted death rate dropping from 4.9 per 100,000 in 1975–1979 to 2.3 per 100,000 in 2010–2019. When stratified by sex, male patients consistently exhibited higher age-adjusted cancer mortality rates than did female patients. The highest age-adjusted cancer mortality rates were consistently observed in the 15–19 years age group, followed by the 5–9 years and 10–14 years age groups.

3. **Survival Trends by SEER Stage**:
   - **Response**: We acknowledge the oversight regarding the presentation of survival data by SEER stage.
   - **The following was added to the methods**: Variables included in our multivariable model were: age, recoded in five-year increments; race; sex; SEER stage; decade, representing the time period or year of diagnosis in ten-year increments; and the ICCC.
   - **A new panel was added to figure 5 showing survival per decade**
   - **A new supplementary table showing survival per stage per decade**
4. **JoinPoint Analysis Discrepancy**:
   - **Response**: The apparent discrepancy in the incidence rates of hepatic tumors highlighted by the JoinPoint analysis versus earlier sections is an important observation.
   - **We added the following sentence to our results**: It should be noted that APC reflects the change over the study period and takes in consideration the rate of change (correlates with the slope) and the starting rates (denominator). The rates of hepatic tumors were very low in the first decade, and showed the highest APC, as mentioned above.
5. **Impact of SEER Dataset Versions**:
   - **Response**: This is a very important point. We cannot fully address the evolution of SEER registries in our manuscript, but we analyzed the registries to spot any differences.
   - The three registries contributed differently to our study population: SEER8 only covered patients before 1992, SEER12 covered patients from 1992 to 1999, and all three registries included patients diagnosed after 2000. To create homogenous groups, we selected patients diagnosed after 2000. This resulted in 22,729 patients enrolled in SEER8, 11,610 in SEER12, and 41,932 in SEER17. Patients enrolled in SEER8 were also included in SEER12 and SEER17, and those enrolled in SEER12 were included in SEER17.
   - Focusing on patients diagnosed after 2000, we observed notable differences, particularly in the representation of the Black race (SEER8: 9.9%, SEER12: 6.7%, SEER17: 12%) and the proportion of leukemia cases (SEER8: 25%, SEER12: 31%, SEER17: 27%). A multivariable Cox regression model, which included age, race, sex, stage, ICCC category, and decade of diagnosis, showed that the risk of death was significantly higher for SEER12 (HR: 1.22) and SEER17 (HR: 1.14), with p-values less than 0.001. This finding might be indicative of truly improving survival of cancer patients despite the inclusion of SEER areas in the more recent analysis.
   - We adjusted our methodology to reflect the uses of these registries in our manuscript.

Iyad Sultan, MD
King Hussein Cancer Center
Amman, Jordan
