# Supplementary material for: Trends in childhood cancer: Incidence and survival analysis over 45 years of SEER data
Source: PLoS One. 2025 Jan 3;20(1):e0314592. doi: 10.1371/journal.pone.0314592 (PMC11698462; doi:10.1371/journal.pone.0314592)
Supplement: Attachment — Submitted filename: Rebuttal_16Oct24.docx [file pone.0314592.s004.docx]

10/16/2024

**Manuscript ID:** PONE-D-24-00928R1
**Title:** Trends in Childhood Cancer: Incidence and Survival Analysis Over 45 Years of SEER Data
Authors: Iyad Sultan, Ahmad Alfaar, Yaseen Sultan, Zeena Salman, Ibrahim Qaddoumi.

Dear Dr. Cho-Hao Howard Lee and Reviewers,

We are grateful for the thoughtful and constructive comments provided by the reviewers. We have addressed each comment in detail below and have revised the manuscript accordingly. Please find our point-by-point responses, followed by a summary of the revisions made to the manuscript.

**Reviewer #2 Comments**

1. **Minor Corrections in Results Section:**
   - **Comment:** The data referenced in the last paragraph of the subsection “Factors affecting survival comparing study periods” appears to be associated with Figure 5 rather than Figure 7.

**Response:** Thank you for bringing this to our attention. We have corrected the figure reference in the Results section. We revised the numbering of figures 4, 5, 6, 7 to fit with text. The section mentioned by the reviewer is now referenced as Fig 6. Legends of figures were fixed accordingly.

**Clarification in Supplementary Table 2:**

- **Comment:** It is unclear how there are some patients with data reported for localized and regional stages for leukemias in Supplementary Table 2.

**Response:** We appreciate this observation. Leukemia stages can theoretically be categorized based on the spread of disease outside the bone marrow, which is not appropriate in our opinion. The staging in SEER is provided as-is with no explanation. In the manuscript, We added a footnote for Supplementary Table 2 explaining that the staging of leukemia was provided by the SEER.

**Reviewer #3 Comments**

1. **Age Group Selection:**
   - **Comment:** The age group cut-offs (<1, 1–4, 5–9, 10–14, and 15–19 years) seem arbitrary. Please explain the rationale behind these cut-offs and consider addressing the population between 19 and 20 years old.

**Response:** We would like to thank the reviewer for flagging this confusing age grouping. According to age recoding by the SEER, patients who are 1 day before their 20^th^ birthday are considered 19 years old. Patients in our study indeed cover those who are between 19 and 20 years old. We added a hint in our methodology: All children and adolescents (aged 0–19 years; i.e. below the age of 20) …

2. **Statistical Analysis and Proportional Hazards Assumption:**
   - **Comment:** It is unclear whether the assumptions of the Cox proportional hazards model were evaluated.

**Response:**

Thank you for your comment on evaluating the assumptions of the Cox proportional hazards model in our study. We acknowledge the importance of this issue and provide the following clarifications:

1. Proportional Hazards Assumption: We employed the `cox.zph()` function to assess this assumption by conducting chi-square tests and calculating p-values for each pair of decades (e.g., 2000 to 2009 versus 2010 to 2019) and for each variable, such as diagnosis. The results showed that the p-values were greater than 0.05 for nearly all tests, confirming the model's validity. However, a notable exception was observed between the decades 1980-1989 and 1990-1999 in leukemia patients, where the p-value was 0.0037, indicating significant differences in this specific instance. Despite this anomaly, the overall results affirm the suitability of the model.
2. Linearity and Model Fit: To ensure the linearity of continuous variables, we applied transformations such as the logarithm of survival months. The model's fit was rigorously validated using Cox-Snell residuals and the concordance statistic, both of which indicated a robust fit.
3. Convergence and Stability: We conducted thorough validations to ensure the model's convergence and the reliability of our estimates, confirming the stability and accuracy of our findings.

- The following paragraph was added to methods: This study rigorously assessed the proportional hazards assumption using the cox.zph() function, which tests each covariate within the model for proportional hazards over time, using pairs of decades as categorical predictors alongside clinical variables such as diagnosis type.

**Multicollinearity and Correlation Among Variables:**

- **Comment:** Potential correlations among age, decade, race, and SEER stage may affect the analysis, and multicollinearity should be checked.

**Response:** Our analysis revealed minor multicollinearity among age group and decade variables. The VIFs for race and SEER stage variables were all below 2, indicating low multicollinearity. The Cramér's V statistics indicated weak associations among the variables in question.

Given that the VIF values were below the commonly accepted threshold of 5 and that the variables are clinically significant, we decided to retain all variables in our model. We have now included detailed descriptions of these analyses in the Methods and Results sections of the manuscript (see Methods, page X; Results, page Y) and we included supplementary tables 2 and 3 with VIF values and Cramér's V values, respectively.

The following paragraph was added to methods: To assess multicollinearity among the predictors, we computed the Variance Inflation Factors (VIF) for each variable using [insert software/tools]. VIF values below the threshold of 5 were considered acceptable, indicating low multicollinearity. Additionally, we computed Cramér's V statistics for categorical variables to evaluate associations between the variables.

The following was added to the results: The VIF values for all variables were below 5, with race and SEER stage exhibiting particularly low multicollinearity (VIF < 1.5), indicating that multicollinearity was not a concern in our model. Cramér’s V statistics showed weak associations among the categorical variables, further supporting the absence of multicollinearity concerns.

3. **Data Continuity and Comparability Over Time:**
   - **Comment:** The data spans from 1975 to 2019, during which diagnostic techniques, treatment methods, and record-keeping practices evolved. These changes could impact the continuity and comparability of the data.

**Response:** We fully agree that changes in medical technology, treatment methods, and record-keeping practices over the 45-year span could influence our results. Unfortunately, the SEER database have no details regarding modalities of diagnosis and treatment. Collectively, these changes impacted trends of cancer diagnosis and treatment. Our analysis is admittedly deficient due to the lack of data. We highlighted this in our limitations section “we faced limitations in accessing crucial information about treatment modalities, such as specific chemotherapy regimens or surgical approaches due to the retrospective nature of the study. This limited our ability to thoroughly analyze the impact of different treatments on survival outcomes.”

4. **Limitations of JoinPoint Analysis:**
   - **Comment:** JoinPoint analysis may not fully account for external factors such as policy changes, medical advances, or public health interventions.

**Response:** We acknowledge the limitations of JoinPoint analysis in accounting for external factors that may influence cancer trends. In the revised manuscript, we have added a discussion of these limitations, including the impact of public health interventions, advances in medical technology, and changes in healthcare policies on the trends observed. We added the following paragraph to the end of our discussion
" While this analysis has been a valuable tool in identifying trends in cancer incidence and survival, it does not fully account for external factors that may influence these trends. Public health interventions, advancements in medical technology, and shifts in healthcare policies can significantly affect the patterns observed. Future studies could benefit from integrating these external factors to provide a more comprehensive understanding of cancer trends and outcomes.”

**Journal Requirements: Reference List Update**

We have thoroughly reviewed our reference list and ensured that all citations are complete and correct. No retracted articles have been cited. Should there be any further concerns regarding our references, we are happy to address them in future revisions.

We hope that the changes made address all the reviewers' concerns satisfactorily. We have attached the revised manuscript, including tracked changes, a clean version, and supplementary materials. We appreciate the reviewers' thoughtful feedback and believe that it has significantly improved the manuscript.

Sincerely,

Iyad Sultan, MD
King Hussein Cancer Center
Amman, Jordan
